# Influence of Bariatric Surgery on Endothelial and Glycocalyx Biomarkers in Obesity and Type 2 Diabetes

**DOI:** 10.1210/clinem/dgaf092

**Published:** 2025-02-13

**Authors:** Maja Andersson, Anna Ågren, Peter Henriksson, Håkan Wallén, Anders Thorell

**Affiliations:** Division of Cardiovascular Medicine, Karolinska Institutet, Department of Clinical Sciences, Danderyd Hospital, Stockholm SE-182 88, Sweden; Department of Cardiology, Danderyd University Hospital Corp, Stockholm SE-182 88, Sweden; Division of Cardiovascular Medicine, Karolinska Institutet, Department of Clinical Sciences, Danderyd Hospital, Stockholm SE-182 88, Sweden; Coagulation Unit, Department of Hematology, Karolinska University Hospital, Stockholm SE-171 76, Sweden; Division of Cardiovascular Medicine, Karolinska Institutet, Department of Clinical Sciences, Danderyd Hospital, Stockholm SE-182 88, Sweden; Department of Cardiology, Danderyd University Hospital Corp, Stockholm SE-182 88, Sweden; Division of Cardiovascular Medicine, Karolinska Institutet, Department of Clinical Sciences, Danderyd Hospital, Stockholm SE-182 88, Sweden; Department of Cardiology, Danderyd University Hospital Corp, Stockholm SE-182 88, Sweden; Department of Surgery and Anesthesia, Ersta Hospital, Stockholm SE-116 91, Sweden; Karolinska Institutet, Department of Clinical Sciences, Danderyd Hospital, Stockholm SE-182 88, Sweden

**Keywords:** bariatric surgery, glycocalyx, endothelial dysfunction, hyaluronan, syndecan-1

## Abstract

**Context:**

Bariatric surgery is associated with reduced risk of cardiometabolic disease in obesity and type 2 diabetes (T2D). The mechanisms are not fully understood, but improvement in endothelial dysfunction has been implicated.

**Objective:**

This work aimed to assess endothelial biomarkers before and after surgery.

**Methods:**

A prospective cohort study with 2-year follow-up was conducted at a single center in Stockholm, Sweden. Participants included adults undergoing bariatric surgery, 28 with and 33 without T2D. Intervention included Roux-en-Y gastric bypass preceded by a 2-week low-calorie diet (LCD). Main outcome measures included plasma concentrations of glycocalyx biomarkers (hyaluronan [HA] and syndecan-1), E-Selectin, von Willebrand factor (VWF), and thrombomodulin (TM).

**Results:**

At baseline, patients with diabetes had higher concentrations of E-Selectin (*P* = .041) whereas other biomarkers did not differ between groups. After LCD, E-Selectin, syndecan-1, and VWF were reduced. Two years after surgery, TM was unchanged whereas E-Selectin decreased, geometric mean (CV%) 41 (40) to 24 (61) ng/mL, syndecan-1 from 50 (73) to 38 (81) ng/mL, and VWF from 120 (52) to 103 (45)%, while HA increased from 25 (96) to 40 (78) ng/mL (*P* < .001 for all). E-Selectin initially declined faster in patients with diabetes (*P* < .003); otherwise the biomarker changes did not differ between groups. Variables with the highest predictive value for improvement in biomarkers were decrease in body weight and fat mass and increase in insulin sensitivity (HOMA-IR).

**Conclusion:**

Bariatric surgery is associated with sustained, beneficial alterations in biomarkers of glycocalyx and endothelial function in patients with obesity, both with and without T2D. It is suggested that reduced body weight/fat mass and improved insulin sensitivity are of particular importance for these alterations.

Obesity and type 2 diabetes (T2D) are both conditions associated with an increased risk of cardiometabolic disease and vascular complications (1, 2). The underlying mechanisms are not fully known but seem to involve obesity-associated low-grade inflammation with vascular endothelial dysfunction and development of a prothrombotic state (3-5). The widespread combination of obesity and T2D might be associated with even further endothelial dysfunction and elevated cardiovascular risk (6, 7).

Bariatric surgery, also referred to as metabolic surgery, results in substantial and sustained weight loss, as well as improvements in, or resolution of, comorbidities such as T2D and cardiovascular disease (CVD) (8-10). These pronounced effects both on obesity and T2D offer an opportunity to study the influence of these two conditions on the development of CVD.

The vascular endothelium has many important physiological functions, and impairment of endothelial cells may lead to fibrotic changes of the vascular wall, development of atherosclerosis, and a prothrombotic state (11). Insulin resistance, which is commonly present in obesity, is known to be associated with impairment of endothelial function (12). One essential, but with respect to obesity and T2D less studied, component of the vascular endothelium is the glycocalyx, a protective gel-like barrier coating the endoluminal surface of blood vessels. This barrier is formed by glycosaminoglycans, proteoglycans, and glycoproteins and separates the blood from the endothelial cell surface, thus maintaining vascular homeostasis. Apart from being “antithrombotic,” the glycocalyx has several other functions such as controlling microvascular tone and permeability as well as regulating inflammation (13). It has become clear that the glycocalyx is vulnerable and may be damaged by conditions like hyperglycemia, inflammation, ischemia, and oxidative stress (14), which are “pathophysiologies” implicated both in T2D and obesity. Damage and disruption of the glycocalyx exposes various endothelial cell bound molecules, like E-Selectin, thrombomodulin (TM), and von Willebrand factor (VWF) to the blood. E-Selectin is involved in the recruitment of leukocytes to inflamed regions of blood vessels and may also be involved in the pathophysiology of atherosclerosis (15), and heart failure with preserved ejection fraction (16). TM mediates anticoagulating and anti-inflammatory effects and is released into plasma following endothelial cell perturbation and damage (17). VWF is another established marker of endothelial dysfunction. It is continuously released by endothelial cells and, like TM, VWF concentrations also increase in plasma following endothelial perturbation and damage (18).

To investigate the effects of weight loss on the vascular endothelium, we assessed concentrations of circulating biomarkers of the glycocalyx (hyaluronan [HA] and syndecan-1).

E-Selectin, TM, and VWF in patients with obesity with and without T2D before and until 2 years after bariatric surgery. Furthermore, we hypothesized that patients with obesity and concomitant T2D, thus suffering from 2 conditions associated with endothelial dysfunction, would respond with more pronounced changes in biomarkers of endothelial function compared to patients with obesity alone.

## Materials and Methods

### Study Design

The study was a single-center, prospective, cohort study with a 2-year follow-up. The study commenced in 2015 with the last study visits in 2021.

Patients with obesity aged 18 to 70 years with or without T2D undergoing bariatric surgery between April 9, 2015 and November 13, 2019, at Ersta Hospital, Stockholm, Sweden, were included. Patients were eligible if planned for Roux-en-Y gastric bypass for obesity fulfilling the criteria for surgery stated by the European Association for the Study of Obesity guidelines from 2015 (19). Patients with type 1 diabetes, and/or ongoing treatment with anticoagulants or antiplatelet agents in the form of P_2_Y_12_ receptor inhibitors, were excluded.

The study cohort were, as far as possible, matched for age and sex. The presence or absence of T2D was primarily determined by self-reported medical history, including use of antidiabetic medication. In addition, blood samples for analysis of fasting plasma glucose (FPG) and glycated hemoglobin A_1c_ (HbA_1c_) were obtained at inclusion. If a patient without known T2D had either a basal FPG and/or an HbA_1c_ concentration consistent with possible T2D, an oral glucose tolerance test was performed to determine if they should be classified as having T2D according to the World Health Organization 2011 criteria (20).

**Figure 1.**
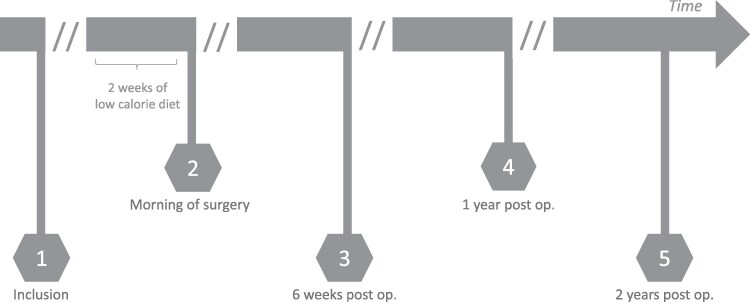
Timeline of the study visits. At all 5 study visits blood samples, anthropometric data, biometric impedance analysis, and health history including medication use were obtained. Visits were in the morning after an overnight fast. Visit 1 = inclusion (baseline), visit 2 = morning of surgery (after low-calorie diet), visit 3 = 6 weeks post surgery, visit 4 = 1 year post surgery, visit 5 = 2 years post surgery.

There were 5 study visits over the 2-year study period: baseline (at inclusion), after a low-calorie diet (LCD) (the morning of surgery), and 6 weeks, 1 year, and 2 years after surgery, respectively (Fig. 1). The time elapsed between inclusion in the study and surgery was on average 13 weeks. At each visit a research nurse recorded anthropometrics including blood pressure, weight, height, and waist/hip circumferences. In addition, the nurse recalled the medical history and any medication use including contraceptive hormones and analgesics. Fat mass was determined using bioelectric impedance analysis (Tanita Inc). Venous blood samples were collected with the patients in the supine position after overnight fasting and at least 20 minutes of rest. For later analyses, 30 mL of blood was collected, whereof 15 mL in citrate tubes, 5 mL in acidified citrate tubes, 5 mL in serum tubes, and 5 mL in heparin tubes and then centrifuged for 20 minutes at 2000*g* at room temperature. Plasma aliquots were subsequently stored at –80 °C.

In accordance with clinical routine, patients were prescribed a mandatory 2-week period of an LCD (Modifast 860 kcal/day) preceding surgery.

### Ethical Aspects

This study was conducted according to the Helsinki Declaration and was approved by the Regional Ehical Review Board in Stockholm (registration No. 2014/1793-31/1). An amendment to the ethical approval was approved by the Swedish Ethical Review Authority (registration No. 2024-05800-02). All patients gave oral and written consent to participate after receiving oral and written information about the study.

### Analyses

All blood chemistry, except biomarkers of endothelial function, were assayed at the local hospital laboratory. Insulin resistance was calculated from glucose and insulin concentrations using the homeostatic model assessment of insulin resistance (HOMA-IR).

Biomarkers for endothelial function were measured on frozen thawed plasma using commercially available enzyme-linked immunosorbent assay (ELISA) kits. Assays were carried out according to the manufacturer's instructions. For HA, E-Selectin, and TM, ELISA kits from R&D Systems Inc were used. For HA, Quantikine ELISA hyaluronan immunoassay (R and D Systems catalog No. DHYAL0) with an inhouse coefficient of variation (CV) of 5.4% was used. For E-Selectin, Human E-Selectin/CD62E (R and D Systems catalog No. DSLE00, RRID:AB_3065116) was used. For TM, Human thrombomodulin/BDCA-3 (R and D Systems catalog No. DTHBD0, RRID:AB_3101767) was used. The Human Syndecan-1 ELISA kit was from Abcam plc (Abcam catalog No. ab46506, RRID:AB_3101768) (inhouse CV 1.9%), and the VWF antigen assay was from Diagnostica Stago, Asserachrom VWF:Ag. (Stago catalog No. 00942, RRID:AB_3101766).

### Statistical Analysis

Due to skewed data distribution, with a need for logarithmic transformation of data before statistical evaluation (discussed later), we used geometric mean and CV (%) for presentation of the data (21).

The linear mixed-models test, which accommodates missing data assuming that data are missing at random, was used to perform statistical inferences. Mixed models are suitable when there are multiple measurements in the same individuals over time and can account for nested structures in the data. Using logarithmically transformed data, the effects over time, between the patient groups (T2D or no diabetes), and the interaction between patient group and time, were evaluated. Based on Akaike's information criteria, either the unstructured covariance matrix or the diagonal covariance matrix was chosen to best represent data.

Paired comparisons within and between groups were performed with the Wilcoxon signed rank and Mann-Whitney *U* test, respectively, and for these analyses no logarithmic transformation of data was required. A *P* value less than 5% was considered statistically significant. Statistical analyses were performed using SPSS software, version 27 (IBM).

To explore whether any variables could predict changes in concentrations of endothelial biomarkers, we performed multivariate data statistical analyses with the orthogonal projection to latent structures (OPLS) model. Traditional regression models have underlying assumptions, mainly that x-variables are independent and exact and that residuals are randomly distributed. In a data set with many variables and a rather small sample size, like our present data set, these assumptions might not be fulfilled. Hence, there was a need for another prediction model. Projection to latent structures (PLS) is one form of regression modeling for multivariate data that can be used. The model we used, OPLS, is a modification of PLS that sorts the data block (x) in two parts, one that is related to the dependent data block y (ie, predictive of y) and one that is orthogonal to y (ie, unrelated to y, also known as “structured noise”). To estimate the influence of the individual x-variables on the OPLS model, variables of importance for projection (VIP) were calculated. The higher the VIP of a certain x-variable, the stronger the influence of this variable in the prediction model and the stronger the correlation (22, 23). A VIP with a value exceeding 1.0 with a CI not including zero was considered important. The OPLS analyses were performed using SIMCA P+, version 15.0.2.0 (Sartorius Stedim Data Analytics AB, MKS).

## Results

### Baseline Characteristics

The patient characteristics at baseline are presented in Table 1. In brief, 61 patients were included, of whom 28 had T2D and 33 did not. Aside from expected differences between groups in variables associated with glucose metabolism, patients with T2D were on average 5 years older, less often current smokers, but more often ex-smokers compared to patients without diabetes. Furthermore, patients with T2D had more often ongoing pharmacological treatment for hypertension, and dyslipidemia (statins) with associated lower total plasma cholesterol levels. Although almost all patients in both groups had insulin resistance (defined as HOMA-IR ≥2.9), it was more pronounced in the group with T2D with a more than 2-fold higher HOMA-IR compared to the group without diabetes.

**Table 1.** Baseline characteristics

	Type 2 diabetes	No diabetes	*P*
**Demographics**			
No.	28	33	
Sex, male/female	14/14	13/20	.41
Age, y	50 (18)	45 (21)	.049
**General medical**			
Weight, kg	113.4 (17)	114.5 (17)	.86
BMI	39.0 (12)	40.0 (11)	.31
Fat mass, kg	47.6 (26)	49.2 (23)	.52
SBP, mm Hg	130 (11)	127 (10)	.29
DBP, mm Hg	83 (10)	85 (11)	.21
**Lab measurements**			
FPG, mmol/L	9.6 (38)	6.1 (10)	<.001
HbA_1c_, %; mmol/mol	7.6; 60 (31)	5.5; 37 (10)	<.001
Insulin, mIE/L	30.2 (98)	21.8 (59)	.046
HOMA-IR	12.9 (105)	5.9 (62)	<.001
Cholesterol, mmol/L	4.4 (21)	5.0 (18)	.007
Triglycerides, mmol/L	1.68 (58)	1.36 (57)	.17
Creatinine, µmol/L	66 (30)	71 (20)	.13

All data, except numbers and sex, are shown as geometric means and coefficients of variation (%). The Mann-Whitney *U* test was used for statistical evaluation of differences between type 2 diabetes and no diabetes.

Abbreviations: BMI, body mass index; DBP, diastolic blood pressure; FPG, fasting plasma glucose; HbA_1c_, glycated hemoglobin A_1c_; HOMA-IR, homeostatic model assessment of insulin resistance; Lab, laboratory; SBP, systolic blood pressure.

Four of the 28 individuals in the T2D group had established macrovascular disease at baseline, of whom 3 had a history of coronary artery disease (myocardial infarction and/or angina pectoris), and one had a history of a transient ischemic attack. No patient in the group without diabetes had a diagnosis of macrovascular disease. In the group with T2D, 5 patients reported diabetes-related microvascular disease (either retinopathy, neuropathy, or nephropathy). The average duration of T2D was 5.2 years (SD 4.8). No statistically significant differences in duration of diabetes were found in patients with macrovascular or microvascular disease compared to diabetes patients free from macrovascular or microvascular disease (*P* = .77 and .11 respectively; Mann Whitney *U* test).

### Effects of Surgery on Anthropometric and Metabolic Variables

As expected, body weight, body mass index (BMI), waist-hip-ratio (WHR), and fat mass were markedly reduced 2 years after surgery (Table 2). In all patients, body weight decreased on average by 27% (31 kg), BMI declined from 40 to 29, WHR decreased by 8%, and total fat mass by 50% (24 kg). There was no difference in the reduction in these weight-related variables between patient groups. However, patients with T2D had consistently slightly higher WHR and lower fat mass throughout the study. Systolic (SBP) and diastolic blood pressure (DBP) were both slightly reduced in both groups.

**Table 2.** Effects of surgery on anthropometric and metabolic variables

	Visit 1	Visit 2	Visit 3	Visit 4	Visit 5	p-1 Time	p-2 Group	p-3 T × G
Weight, kg								
T2D	113 (17)	108 (17)	99 (17)	83 (20)	84 (21)			
No T2D	115 (17)	110 (15)	101 (16)	85 (20)	83 (21)	<.001	.49	.98
BMI								
T2D	39.0 (12)	37.0 (12)	34.1 (14)	28.5 (19)	28.9 (20)			
No T2D	40.0 (11)	38.5 (10)	35.4 (11)	29.5 (14)	28.8 (14)	<.001	.097	.94
Waist, cm								
T2D	123.5 (10)	120.9 (9)	112.4 (10)	98.4 (13)	97.6 (14)			
No T2D	121.9 (10)	117.8 (10)	111.5 (11)	98.4 (12)	95.0 (14)	<.001	.29	.97
WHR								
T2D	1.01 (11)	1.01 (8)	0.99 (11)	0.94 (8)	0.92 (9)			
No T2D	0.97 (10)	0.95 (11)	0.95 (11)	0.92 (9)	0.90 (10)	<.001	.001	.71
Fat mass, kg								
T2D	47.6 (26)	43.7 (26)	36.4 (26)	22.7 (47)	23.7 (49)			
No T2D	49.2 (23)	47.1 (19)	40.5 (23)	26.5 (29)	24.6 (31)	<.001	.027	.84
SBP, mm Hg								
T2D	130 (11)	122 (11)	123 (12)	119 (13)	129 (13)			
No T2D	127 (10)	123 (10)	121 (12)	121 (14)	124 (16)	.012	.34	.80
DBP, mm Hg								
T2D	83 (10)	79 (12)	77 (11)	77 (13)	81 (15)			
N T2D	85 (11)	80 (12)	79 (11)	80 (9)	82 (13)	.006	.081	.98
FPG, mmol/L								
T2D	9.6 (38)	7.3 (34)	7.5 (56)	6.2 (38)	6.2 (19)			
No T2D	6.1 (10)	5.6 (11)	5.6 (11)	5.4 (12)	5.4 (10)	<.001	<.001	<.001
HbA_1c_, %;mmol/mol								
T2D	7.6; 60 (31)	7.3; 56 (28)	6.3; 45 (31)	5.9; 41 (40)	5.9; 41 (23)			
No T2D	5.5; 37 (10)	5.4; 35 (13)	5.3; 34 (14)	5.3; 34 (8)	5.3; 34 (11)	<.001	<.001	<.001
Insulin, mIE/L								
T2D	30.2 (98)	20.2 (83)	14.3 (60)	10.0 (68)	10.2 (69)			
No T2D	21.8 (59)	14.7 (50)	13.7 (46)	8.4 (103)	8.2 (36)	<.001	.045	.18
HOMA-IR								
T2D	12.9 (105)	6.6 (59)	4.8 (128)	2.8 (84)	2.8 (84)			
No T2D	5.9 (62)	3.7 (54)	3.4 (58)	2.0 (128)	2.0 (40)	<.001	<.001	.042
Cholesterol, mmol/L								
T2D	4.4 (21)	3.5 (23)	3.7 (18)	4.1 (21)	4.3 (20)			
No T2D	5.0 (18)	4.4 (27)	4.2 (20)	4.4 (19)	4.4 (18)	<.001	<.001	.16
LDL, mmol/L								
T2D	2.4 (34)	1.7 (40)	1.8 (27)	2.1 (34)	2.2 (33)			
No T2D	3.1 (23)	2.6 (39)	2.3 (27)	2.3 (31)	2.3 (32)	<.001	.006	.013
HDL, mmol/L								
T2D	1.1 (26)	1.1 (34)	1.1 (25)	1.4 (21)	1.5 (30)			
No T2D	1.2 (24)	1.1 (23)	1.2 (29)	1.4 (25)	1.5 (26)	<.001	.024	.94
TGs, mmol/L								
T2D	1.68 (58)	1.30 (41)	1.36 (58)	1.10 (52)	1.08 (31)			
No T2D	1.36 (57)	1.20 (47)	1.32 (58)	1.09 (56)	1.02 (63)	<.001	.34	.25
LEU, 10^9^/L								
T2D	7.37 (27)	7.06 (29)	6.38 (26)	6.39 (25)	6.17 (25)			
No T2D	7.20 (24)	6.61 (22)	6.24 (23)	6.08 (21)	6.24 (17)	<.001	.27	.91

Data are shown as geometric means and coefficients of variation (%). Mixed-models analysis was used for statistical evaluation of effects of time (p-1), group (p-2), and interaction between time and group (T × G; p-3). E-Selectin, hyaluronan, and syndecan-1 concentrations are ng/mL. Visit 1 = baseline (inclusion), visit 2 = after low-calorie diet (morning of surgery), visit 3 = 6 weeks post surgery, visit 4 = 1 year post surgery, visit 5 = 2 years post surgery; for further information, see Fig. 1.

**Abbreviations:** DBP, diastolic blood pressure; FPG, fasting plasma glucose; HbA_1c_, glycated hemoglobin A_1c_; HOMA-IR, homeostatic model assessment of insulin resistance; LEU, leukocytes; SBP, systolic blood pressure; T2D, type 2 diabetes; TGs, triglycerides; TM, thrombomodulin (pg/mL); VWF, von Willebrand factor antigen (%); WHR, waist-hip-ratio.

HOMA-IR as well as concentrations of glucose, HbA_1c_, and insulin improved in both groups (*P* < .001); statistically significantly more so in patients with T2D (*P* < .001 for the interaction term for all except for insulin, where the decrease did not differ between groups). Lipid profiles were also improved in both groups, with a more pronounced reduction in low-density lipoprotein levels in patients without diabetes.

### Type 2 Diabetes Status and Antidiabetic Medications After Surgery

**Figure 2.**
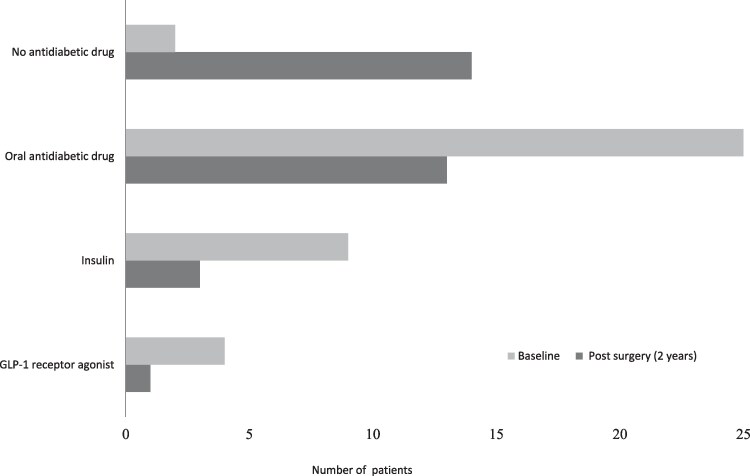
Use of antidiabetic drugs by patients with T2D, comparing baseline with 2 years post surgery. One patient can be represented in more than one category (ie, use of both insulin and an oral antidiabetic drug).

Two years after surgery, 13 out of 28 patients with T2D at baseline were in complete remission, defined as HbA_1c_ less than 6.5% (<48 mmol/mol) at 2 consecutive measurements over 1 year without any pharmacological antidiabetic medication (24). Half of the patients (n = 14) were not in complete remission and in 1 patient, data regarding medication use at the 2-year follow-up were lacking. Of the 13 patients that were still on antidiabetic drug treatment at 2 years after surgery, all but 1 patient had either reduced their doses (n = 6), or their number of medications (n = 2), or both (n = 4). Use of various types of antidiabetic medication before and after surgery is shown in Fig. 2.

### Endothelial Biomarkers at Baseline

At baseline only E-Selectin, of the endothelial biomarkers studied, differed between the two groups, being significantly higher in patients with T2D (46.6 [37] vs 36.5 [40] ng/mL; *P* = .041) (Table 3).

**Table 3.** Effects of surgery on endothelial biomarkers

	Visit 1	Visit 2	Visit 3	Visit 4	Visit 5	p-1 Time	p-2 Group	p-3T × G
E-Selectin								
T2D	46.6 (37)	35.6 (47)	26.6 (52)	22.8 (53)	23.5 (59)			
No T2D	36.5 (40)	28.7 (47)	25.8 (49)	23.5 (53)	24.8 (63)	<.001	.47	.003
Hyaluronan								
T2D	27.4 (87)	23.8 (55)	35.3 (85)	39.4 (57)	41.2 (68)			
No T2D	23.6 (104)	23.9 (104)	35.7 (82)	37.9 (73)	39.0 (85)	<.001	.69	.90
Syndecan-1								
T2D	46.3 (78)	41.0 (54)	30.4 (110)	39.0 (94)	35.6 (109)			
No T2D	52.4 (70)	47.6 (45)	39.2 (62)	45.2 (100)	41.1 (47)	<.001	.21	.55
VWF								
T2D	125.7 (43)	111.7 (35)	108.3 (56)	98.8 (41)	96.9 (36)			
No T2D	115.3 (60)	106.6 (56)	115.2 (48)	100.3 (46)	103.0 (49)	<.001	.92	.42
TM								
T2D	4117 (39)	4087 (33)	4025 (39)	4169 (32)	3900 (30)			
No T2D	4198 (18)	4068 (20)	4167 (21)	4049 (23)	4231 (28)	.99	.48	.80

Data are shown as geometric means and coefficients of variation (%). Mixed-models analysis was used for statistical evaluation of effects of time (p-1), according to group (p-2), and interaction between time and group (T × G; p-3). E-Selectin, Hyaluronan, and Syndecan-1 concentrations are ng/mL. Visit 1 = baseline, visit 2 = after low-calorie diet, visit 3 = 6 weeks post surgery, visit 4 = 1 year post surgery, visit 5 = 2 years post surgery; for further information see Fig. 1.

**Abbreviations:** T2D, type 2 diabetes; TM, thrombomodulin (pg/mL); VWF, von Willebrand factor antigen (%).

### Effects of Low-Calorie Diet and Surgery on Endothelial Biomarkers Over 2 Years

In response to the preoperative 2-week period of an LCD, as presented in Table 3, E-Selectin, syndecan-1, and VWF concentrations decreased significantly in both groups while TM and HA were unchanged. The group with T2D had a more rapid decline in E-Selectin initially, during the LCD, and during the early postoperative period (6 weeks), resulting in a statistically significant interaction term in the mixed-model analysis (*P* = .003) when comparing the groups. Over the 2-year follow-up, E-Selectin, syndecan-1, and VWF decreased significantly, whereas HA increased significantly (*P* < .001 for all). In contrast, there were no statistically significant alterations in plasma concentrations of TM over the same period. The effects on endothelial biomarkers after surgery over the follow-up period were similar in both groups.

### Multivariate Regression Analysis of Variables Predicting Changes in Endothelial Biomarkers 2 Years After Surgery (All Patients)

**Figure 3.**
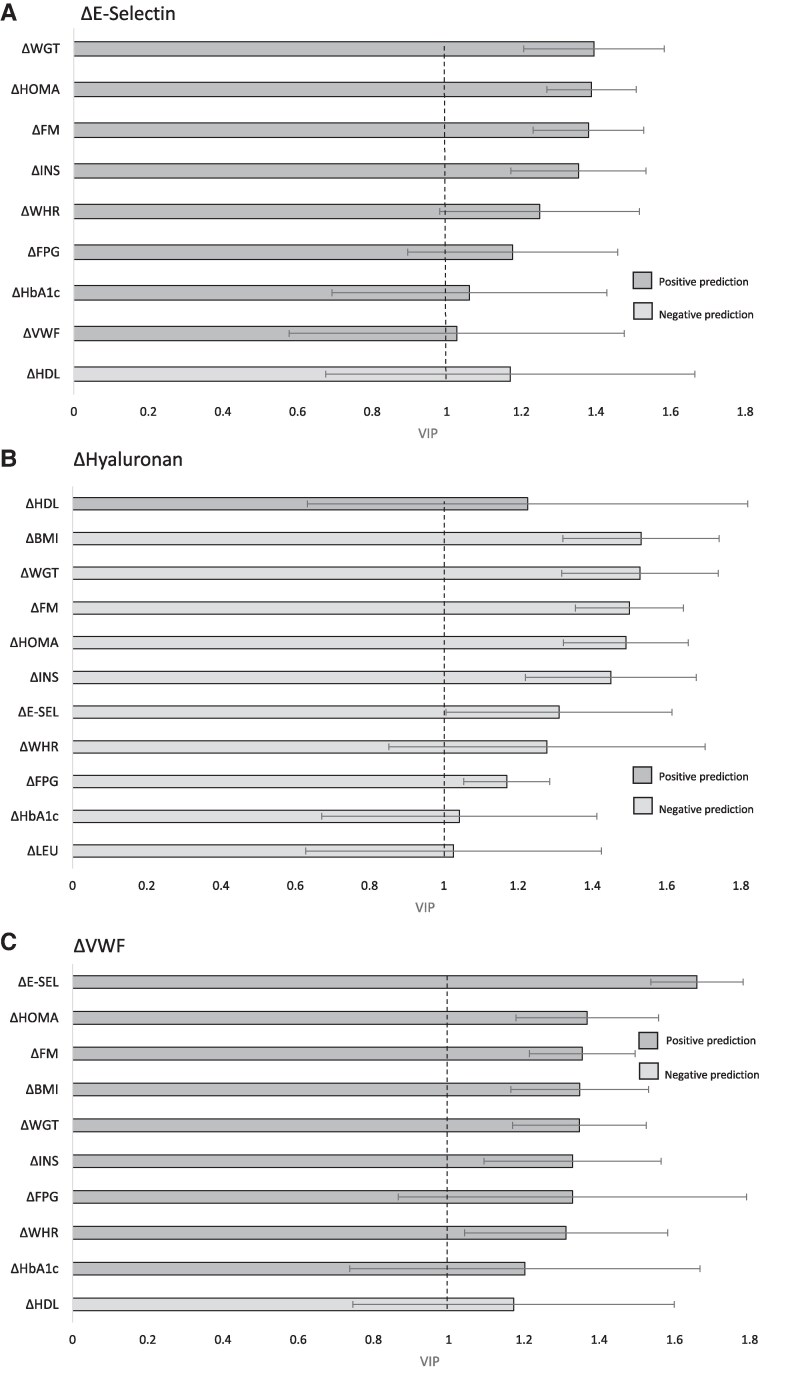
Regression modeling with orthogonal projections to latent structures (OPLS) depicting the variables of importance in projection (VIPs) that predict changes in A, E-selectin; B, hyaluronan; and C, von Willebrand factor 2 years after surgery, respectively. A VIP of 1.0 or above is considered statistically significant. Error bars are 95% CIs. Dark gray bars represent positive associations; light gray bars represent negative associations. Δ represents the relative difference of the variable 2 years after surgery compared to baseline. E-SEL, E-Selectin; FM, fat mass; FPG, fasting plasma glucose; HbA_1c_, glycated hemoglobin A_1c_; HDL, high-density lipoprotein; HOMA, HOMA-IR homeostatic model of insulin resistance; INS, insulin; LEU, leukocytes; VWF, von Willebrand factor; WGT, weight; WHR, waist-hip-ratio.

Metabolic and clinical variables known to be influenced by bariatric surgery were analyzed using OPLS modeling for prediction of changes in endothelial biomarkers at the 2-year follow-up. The VIPs that turned out to be statistically significant for prediction are shown in Fig. 3. Weight reduction, reduction in fat mass, and reduction in insulin sensitivity (HOMA-IR) were all encountered among the 5 most important VIPs for changes in E-Selectin, HA, and VWF. Of note, for HA, which increased following surgery, the associations with changes in weight, fat mass, and HOMA-IR were inverse.

Regarding syndecan-1, only HOMA-IR and FPG had a VIP above 1.0, and with wide CIs: VIP(HOMA-IR): 1.09 (95% CI, 0.16-2.01), VIP(FPG): 1.03 (95% CI, 0.33-1.72).

## Discussion

In this study, we report marked alterations in concentrations of 2 circulating biomarkers of the vascular endothelial glycocalyx, HA, and syndecan-1, 2 years after bariatric surgery in patients with obesity, with as well as without T2D. The effects of bariatric surgery on biomarkers of the glycocalyx are novel findings that have not, to our knowledge, been reported previously.

HA is a major component of the endothelial glycocalyx and a ubiquitous component of the extracellular matrix (25). At baseline, HA concentrations in plasma were within the expected range (26) and increased at 2 years compared to baseline. We used an assay that measures HA of higher molecular weight, above 30 to 35 kDa, but does not detect low-molecular-weight HA. It is therefore unlikely that we measured the low-molecular-weight fractions of HA ascribed to act as so-called “damage-associated molecular patterns” (DAMPs), related to proinflammatory effects (27). This suggests that loss of fat mass may increase the abundance of middle- to high-molecular-weight HA in plasma, with potential protective effects on the vascular endothelium and other tissues (28). As obesity with excessive fat mass is associated with an elevated production of reactive oxygen species (ROS) (29), and since ROS degrades HA (30), a reduced production of ROS following loss of fat mass with reduced degradation of HA may hypothetically explain why HA concentrations increase following bariatric surgery. Of note, since HA might appear in plasma from multiple sources, the proportion of circulating HA emanating from the endothelial glycocalyx is difficult to estimate. It seems reasonable, however, to assume that the increase in circulating HA observed herein, at least in part reflects increased vascular endothelial production of HA considering the vast spatial distribution of HA containing endothelium within the body (31).

In contrast to the increased HA concentrations, we observed decreased concentrations of circulating syndecan-1 after weight loss. In its cell-surface bound form, syndecan-1 constitutes an essential part of the glycocalyx that binds glycosaminoglycans like heparan sulphate and chondroitin sulphate. Syndecan-1 also serves as a receptor for growth factors, cytokines, adhesion molecules, and proteases, and as such it mediates various intracellular effects through its cytoplasmic domain (32). The proteolytical release of syndecan-1 with shedding into plasma occurs in response to inflammation (33). There are some studies indicating that syndecan-1 concentrations are elevated both in obesity and T2D (34, 35). The reduction in syndecan-1 concentration after weight loss in our study is in agreement with reduced perturbation of the vascular endothelium.

The reduction in soluble E-Selectin further supports previous findings that surgically induced weight loss confers reduced activation of the vascular endothelium (36). This selectin is, like syndecan-1, expressed on the endothelium and shed into plasma and its soluble form is considered to reflect systemic activation of the endothelium (37). The increased expression and release of E-Selectin is stimulated by proinflammatory cytokines such as tumor necrosis factor α and interleukin-1β, both of which are known to decrease following bariatric surgery (38). Interestingly, elevations in soluble E-Selectin in young adults has recently been shown to be associated with more unfavorable indices of cardiac function later in life, and high BMI was one of the factors associated with higher E-Selectin levels (16). In a large cohort study, increased E-Selectin concentrations could, independently of obesity, predict onset of T2D (39). Our data, indicating an improved endothelial function up to 2 years after bariatric surgery, is in line with the idea that this intervention may have long-term beneficial effects on risk of developing CVD. Speculatively, this may at least partly be mediated by beneficial effects on the vascular endothelium. Of note, the changes in markers of endothelial function noted in our study coincide with previous reports on long-term improvements in functional vascular parameters after bariatric surgery (beyond 1 year), such as flow-mediated dilatation and hyperemia, reflecting endothelial function and that in large studies on CVD predict outcomes (40-42).

Plasma concentrations of VWF decreased over time in this study, which is consistent with some previous reports (43). This, together with the effects on both glycocalyx biomarkers and E-Selectin, further supports our hypothesis that the protective cardiovascular effects seen after surgically induced weight loss could be partially explained by a “healthier” endothelium.

Concentrations of E-Selectin, syndecan-1, and VWF decreased significantly following 2 weeks of preoperative fasting (LCD). This was observed together with statistically significant reductions in weight, blood pressure, blood lipids, and insulin concentrations. However, no effect on HA concentrations was seen after LCD, in contrast to E-Selectin, syndecan-1, and VWF. Indeed, diet-induced improvement in endothelial function has been reported in short-term studies (44), corroborating our findings. The underlying mechanisms of diet-induced effects on vascular endothelium are, however, complex, and our study was not designed to further explore this in detail. The increased HA concentrations seen over time might reflect a more long-term beneficial effect of the surgical intervention.

Circulating concentrations of TM were not significantly affected in our study. There are few previous studies examining the effect of bariatric surgery on soluble TM and with ambiguous results; both reduced (45) and unchanged (46) TM concentrations have been reported. We have no explanation for these discrepancies and cannot fully exclude a type II error in this respect in our study.

This study was not designed to prove causation of bariatric surgery on concentrations of endothelial biomarkers. However, the multivariate prediction model showed that the effects seen on E-Selectin, VWF, and HA were foremost associated with the weight loss, reduction in fat mass, and increased insulin sensitivity. For syndecan-1 these effects were less distinct in the prediction model, with weak associations observed for decreased fasting glucose and improved insulin sensitivity. The prediction model supports our hypothesis that improved endothelial and glycocalyx function after bariatric surgery is a treatment effect dependent primarily on weight loss and fat mass reduction, to which improved glucose homeostasis also may contribute.

Apart from E-Selectin, no differences were observed in circulating biomarkers representing endothelial function when we compared the effects of surgery in patients with and without T2D over time. This may have several explanations; one could be that although we used strict criteria for diagnosing diabetes, the two patient groups were too similar with respect to aberrations in glucose metabolism to detect differences in response to the surgical intervention. Of note, 90% of patients without manifest T2D were insulin resistant, and more than half of those patients had prediabetes (impaired fasting glucose). On the other hand, HOMA-IR was twice as high in patients with T2D, suggesting that the disturbances in glucose control were, in fact, distinct. Another explanation for the lack of differences between the groups could therefore be that obesity and its associated pathophysiology, regardless of diabetes status, might be the front runner for effects on the endothelium, and that the presence of T2D has less effect on endothelial function in the setting of severe obesity. E-Selectin showed a statistically significant difference between groups where patients with diabetes had a higher baseline concentration and decreased more rapidly following an LCD and during the early postoperative period (6 weeks). This is an interesting finding but since the aim of this study was to examine the long-term effects of surgically induced weight loss on the endothelium, we refrain from drawing any firm conclusions from these early differences between groups.

In summary, bariatric surgery was associated with sustained alterations in circulating biomarkers of glycocalyx and endothelial function both in obesity and T2D. However, the relative importance of reduced fat mass vs improvement of diabetes and insulin sensitivity for these changes needs to be addressed in future studies.

### Limitations

There are limitations in our study. The two study groups, diabetes and nondiabetes patients, differed with respect to pharmacological treatment and clinical characteristics other than the diabetes diagnosis. This may have influenced the results of the study.

It was not possible to clearly distinguish the effect of surgery from some contributing effects of pharmacological treatment with antihypertensive or lipid-lowering drugs. Regarding antidiabetic drugs, most patients with T2D were either completely off antidiabetic treatment or had markedly reduced their dosage after surgery. Therefore, the effect of the surgery would, if anything, have been underestimated. Further, it could be assumed that the effect of any remaining pharmacological antidiabetes treatment was relatively minor.

In the group without diabetes a vast majority had either prediabetes or insulin resistance. To recruit enough patients with obesity without prediabetes proved to be difficult, and one could argue that such patients would constitute a subgroup that would reduce the external validity of the study. It cannot be excluded that the lack of differences for most endothelial variables between patients with and without diabetes might at least partly be due to the limited differences in glucose homeostasis, even though we found clear-cut and statistically significant differences in FPG, HbA_1c_, and HOMA-IR between the groups. A control group including lean individuals would have been preferable, but for obvious reasons this was not possible.

The method used for measurements of HA does not allow more precise size discrimination of HA molecules, limiting the possibility to in more detail determine changes in low-molecular-weight species following surgery.

The sample size might have been too small to detect minor differences for some endothelial biomarkers between the groups. The reason for the sample size being smaller than initially planned was that recruitment of male patients without diabetes turned out to be harder than expected, possibly because males might be less prone to be referred for surgery. Some loss to follow-up of data occurred.

### Strengths

The study duration, design, and the characterization of the cohort together with the standardized intervention are the main strengths of this study. This is, to the best of our knowledge, one of the largest studies comparing blood biomarkers of endothelial function in patients with T2D and obesity vs obesity alone before and after bariatric surgery. It entails a comparatively long follow-up including a well-characterized cohort encompassing details regarding comorbidities and medication use. The study design in which the participants constitute their own control, before and after weight loss, can reduce some of the bias that arises in cross-sectional studies where differences in biomarkers between patients with obesity and those with normal body weight are compared.

## Data Availability

Restrictions apply to the availability of some or all data generated or analyzed during this study to preserve patient confidentiality. The corresponding author will on request detail the restrictions and any conditions under which access to data may be provided.

## Abbreviations

CV: coefficient of variation
CVD: cardiovascular disease
DBP: diastolic blood pressure
ELISA: enzyme-linked immunosorbent assay
FPG: fasting plasma glucose
HA: hyaluronan
HbA_1c_: glycated hemoglobin A_1c_
HOMA-IR: homeostatic model assessment of insulin resistance
LCD: low-calorie diet
LEU: leukocytes
OPLS: orthogonal projection to latent structures
ROS: reactive oxygen species
SBP: systolic blood pressure
T2D: type 2 diabetes
TGs: triglycerides
TM: thrombomodulin
VIP: variables of importance in projection
VWF: von Willebrand factor antigen
WHR: waist-hip-ratio
